# Regulatory T Cells Increase After rh-MOG Stimulation in Non-Relapsing but Decrease in Relapsing MOG Antibody-Associated Disease at Onset in Children

**DOI:** 10.3389/fimmu.2021.679770

**Published:** 2021-06-16

**Authors:** Philippe Horellou, Aliénor de Chalus, Laetitia Giorgi, Carole Leroy, Pascale Chrétien, Salima Hacein-Bey-Abina, Christine Bourgeois, Xavier Mariette, Ché Serguera, Roger Le Grand, Kumaran Deiva

**Affiliations:** ^1^ Université Paris-Saclay, CEA, INSERM UMR 1184, Le Kremlin Bicêtre, France; ^2^ Assistance Publique-Hôpitaux de Paris, Paris-Saclay University Hospitals, Bicêtre Hospital, Pediatric Neurology Department, Le Kremlin Bicêtre, France; ^3^ Clinical Immunology Laboratory, Groupe Hospitalier Universitaire Paris-Sud, Hôpital Kremlin-Bicêtre, Assistance Publique-Hôpitaux de Paris, Le-Kremlin-Bicêtre, France; ^4^ Université de Paris, CNRS, INSERM, UTCBS, Unité des technologies Chimiques et Biologiques pour la Santé, Paris, France; ^5^ Department of Rheumatology, Hôpital Bicêtre, Assistance Publique–Hôpitaux de Paris (AP-HP), Le Kremlin-Bicêtre, France; ^6^ Institut du Cerveau (ICM), Hôpital Pitié-Salpêtrière, Paris, France; ^7^ National Referral Center for Rare Inflammatory and Auto-Immune Brain and Spinal Diseases (MIRCEM), Pediatric Neurology Department, Hôpital Bicêtre, AP-HP, Le Kremlin Bicêtre, France

**Keywords:** neuroinflammation, auto-immune diseases, acquired demyelinating syndromes (ADS), myelin oligodendrocyte glycoprotein, multiple sclerosis, regulatory T lymphocytes

## Abstract

**Background:**

Myelin oligodendrocytes glycoprotein (MOG) antibody-associated disease (MOGAD) represent 25% of pediatric acquired demyelinating syndrome (ADS); 40% of them may relapse, mimicking multiple sclerosis (MS), a recurrent and neurodegenerative ADS, which is MOG-Abs negative.

**Aims:**

To identify MOG antigenic immunological response differences between MOGAD, MS and control patients, and between relapsing *versus* non-relapsing subgroups of MOGAD.

**Methods:**

Three groups of patients were selected: MOGAD (n=12 among which 5 relapsing (MOGR) and 7 non-relapsing (MOGNR)), MS (n=10) and control patients (n=7). Peripheral blood mononuclear cells (PBMC) collected at the time of the first demyelinating event were cultured for 48 h with recombinant human (rh)-MOG protein (10 μg/ml) for a specific stimulation or without stimulation as a negative control. The T cells immunophenotypes were analyzed by flow cytometry. CD4^+^ T cells, T helper (Th) cells including Th1, Th2, and Th17 were analyzed by intracellular staining of cytokines. Regulatory T cells (T_regs_, Foxp3^+^), CD45RA^-^Foxp3^+^ T_regs_ and subpopulation naive T_regs_ (CD45RA^+^Foxp3^int^), effector T_regs_ (CD45RA^-^Foxp3^high^) and non-suppressive T_regs_ (CD45RA^-^Foxp3^int^) proportions were determined.

**Results:**

The mean onset age of each group, ranging from 9.9 to 13.8, and sex ratio, were similar between MOGR, MOGNR, MS and control patients as analyzed by one-way ANOVA and Chi-square test. When comparing unstimulated to rh-MOG stimulated T cells, a significant increase in the proportion of Th2 and Th17 cells was observed in MOGAD. Increase of Th17 cells was significant in MOGNR (means: 0.63 ± 0.15 *vs.* 1.36 ± 0.43; Wilcoxon-test *p* = 0.03) but not in MOGR. CD4^+^ T_regs_ were significantly increased in MOGNR (means: 3.51 ± 0.7 *vs.* 4.59 ± 1.33; Wilcoxon-test *p* = 0.046) while they decreased in MOGR. CD45RA^-^Foxp3^+^ T_regs_ were significantly decreased in MOGR (means: 2.37 ± 0.23 *vs.* 1.99 ± 0.17; paired t-test *p* = 0.021), but not in MOGNR. MOGR showed the highest ratio of effector T_regs_/non suppressive-T_regs,_ which was significantly higher than in MOGNR.

**Conclusions:**

Our findings suggest that CD4^+^ Th2 and Th17 cells are involved in the pathophysiology of MOGAD in children. The opposite response of T_regs_ to rh-MOG in MOGNR, where CD4^+^ T_regs_ increased, and in MOGR, where CD45RA^-^Foxp3^+^ T_regs_ decreased, suggests a probable loss of tolerance toward MOG autoantigen in MOGR which may explain relapses in this recurrent pediatric autoimmune disease.

## Introduction

Pediatric acquired demyelinating syndromes (ADS) are rare immune-mediated acute demyelinating disorders of the central nervous system (CNS) with an incidence of 0.6 to 1.6 for 100,000 children per year in western countries (1–3). Myelin oligodendrocytes glycoprotein (MOG) antibodies (Abs) are found in about 25% of pediatric ADS (4) now referred to as MOG antibody-associated disease (MOGAD). MOG protein represents only 0.05% of myelin proteins and is expressed exclusively on the outer surface of the myelin sheath and the plasma membrane of oligodendrocytes. Its cell surface location makes it accessible to immune reactions (5) becoming a target of autoimmune responses that cause inflammation and CNS demyelination (6, 7). The course of MOGAD can be either non-relapsing (MOGNR) or relapsing (MOGR). Unlike MOGAD, multiple sclerosis (MS) patients do not have MOG-Abs (8–10). MS is an ADS characterized by the recurrence of demyelination episodes resulting in subsequent neurological damage. Both MOGAD and MS have T and B lymphocytes infiltration in the brain, but in MS, CD8^+^ T cell and B cell infiltration is higher (11) than in MOGAD where CD4^+^ T cell infiltration is predominant (12). There are evidence that autoreactive CD4^+^ T-cells are involved in both MOGAD and MS pathogenesis, but further research is required to understand their role in the disease onset and evolution (13, 14).

In experimental autoimmune encephalomyelitis (EAE) in rodents, both cell-mediated and humoral immune responses are involved in brain inflammation and demyelination (15, 16), sustained by a T/B cell cooperation as studied using transgenic mice with MOG-specific T and B cell receptors (17–21). *In vitro* experiments in humans have shown that patient sera containing MOG-Abs, activate the complement pathway (22–24), induce natural killer cells and antibody-dependent cell-mediated cytotoxicity (6), and can result in the disruption of the oligodendrocyte cytoskeleton (25, 26).

A non-human primate model of EAE in cynomolgus macaques was developed by sensitization with recombinant human (rh)-MOG, emulsified in incomplete Freund’s adjuvant (IFA), which induced a disease similar to MOGAD in children (27). Interestingly, in this model, it was recently shown that the intradermal routing of MOG into resident dendritic cell asialoglycoprotein receptor (DC-ASGPR)^+^ cells using recombinant antibody DC-ASGPR fused to MOG prevented the breach of immune tolerance against MOG after rh-MOG/IFA sensitization. Phenotyping of blood lymphocytes indicated that only the control animals had an increase activation of CD4^+^ T cells in the days preceding the onset of EAE. In contrast, animals treated with anti-DC-ASGPR-MOG had an increase in MOG-specific T_regs_ upon rh-MOG/IFA re-administration (28).

These results prompted us to evaluate the response of CD4^+^ T cells to rh-MOG stimulation *in vitro* from children with different forms of pediatric ADS. We compared the CD4^+^ T cells immunological phenotypes of MOGAD patients to MS and control patients with non-inflammatory neurological diseases, and assessed the cells functional responses after stimulation with rh-MOG protein *in vitro*. We focused our analysis on the three main T helper (Th) cells corresponding to three lineages of CD4^+^ lymphocytes triggered upon antigenic activation. These CD4^+^ T cells are referred to as Th type-1 (Th1), Th type-2 (Th2) or Th type-17 (Th17) cells, according to their phenotype. Th1 cells produce interferon-γ (IFN-γ) and tumor necrosis factor-alpha (TNF-α) and are effective against intracellular bacteria and viruses, but are also involved in autoimmune diseases. Th2 cells secrete interleukin-4 (IL-4), -5, -10 and -13, which up-regulate antibody production through B cells activation. Th17 cells secrete IL-17 and TNF-α and are involved in tissue inflammation, activation of neutrophils and in autoimmunity. We then studied regulatory T (T_reg_) cells that secrete IL-10 and transforming growth factor-beta (TGF-β), which modulate Th cell activity and suppress some of their functions, inducing tolerance to antigens. We then subdivided the MOGAD group into non-relapsing MOGAD (MOGNR) and relapsing MOGAD (MOGR) to further evaluate relations between relapse and immunological response to rh-MOG.

## Materials and Methods

### Patients and Controls

Twenty-two children ≤ 18 years old, from the French cohort KIDBIOSEP, followed for a first demyelinating episode in the national reference center for rare inflammatory brain and spinal diseases at Bicêtre Hospital, from January 2011 to May 2018, were included. ADS was defined as an acute neurological deficit lasting more than 24 h in the CNS, affecting the optic nerve, brain, cerebellum, brainstem and/or spinal cord associated with T2 lesions on magnetic resonance imaging (MRI). Relapse was defined as a new episode of CNS demyelination at least 1 month after the first episode or 3 months after the first episode if the first attack is an acute demyelinating encephalomyelitis (ADEM), and lasting for at least 24 h in the absence of fever or infection. The MS diagnosis was made according to the 2013 IPMSSG criteria and the revised 2010 MacDonald criteria. We classified our patients into 3 groups: MOGAD patients, including non-relapsing (MOGNR) and relapsing (MOGR) subgroups based on the progression of the disease after the blood sampling, ADS without MOG-Ab corresponding to MS patients and control patients (CTRL). Seven control patients were included from pediatric neurology department of Bicêtre Hospital for non-inflammatory neurological diseases, such as intracranial hypertension (n=1), psychosomatic syndromes (n=2), genetical peripheral neuropathies (n=2), psychomotor developmental delays (n=1) or stroke (n=1), for which blood samples were performed for diagnosis. Demographic data of included children are presented in **Table 1**.

**Table 1:** Demographic data of included children.

	MOGRn=5	MOGNRn=7	MSn=10	Controlsn=7	*p* value
Female (n (%))	5 (100)	2 (29)	4 (40)	2 (29)	0.051
Age at onset (mean, years ± SD)	9.9 ± 2.4	10.2 ± 5.2	13.8 ± 2.2	11 ± 3.2	0.114
Presentation (n (%))					
Optic neuritis	2 (40)	1 (14)	3 (30)		0.594
Transverse myelitis	2 (40)	4 (57)	0		0.025
ADEM	0	2 (29)	0		0.48
Rhombencephalitis	1 (20)	1 (14)	2 (20)		0.9
Long tract involvement	0	0	6 (60)		0.007
Others	0	0	1 (10)		0.454
Follow-up time (mean, years ± SD)	4.9 ± 2.2	1.9 ± 2.2	2.3 ± 2	0.5 ± 0.5	0.005

Data are expressed as mean ± standard deviation (SD) unless otherwise stated. MOGR: relapsing MOGAD, MOGNR: non-relapsing MOGAD. MS: multiple sclerosis patients. Controls: other non-inflammatory neurological disorder patients. For age at onset and follow-up time, one-way ANOVA p values are given. For gender and presentation, the Chi-square p value are given, except for ADEM, Rhombencephalitis and Others for which only Fisher’s exact test could be used.

### Ethics

This study complied fully with French national and local ethics committee guidelines. The national cohort of first demyelinating episode “Kidbiosep 2004” (No. 910506) was authorized by the Commission Nationale de l’Informatique et des Libertés and the Comité de Protection des Personnes of Paris-Saclay University. An informed consent form was signed by parents of each included child.

### Blood Samples

Blood samples were collected on heparinized tubes from all patients at their first demyelinating event, within the first 3 months and before starting immunosuppressive or immunoregulatory therapy. For all patients, PBMCs were isolated by Ficoll density gradient centrifugation. Briefly, heparinized blood was centrifuged at 700 × g for 15 min and the top layer containing plasma was removed, transferred in cryovials and stored at -20°C. The remaining blood was diluted with an equal volume of isosmotic 0.9% wt/vol NaCl solution layered over 15 ml of the Ficoll-Paque PLUS (GE Healthcare). Gradients were centrifuged at 700 × g for 30 min at room temperature. The PBMC interface was removed by pipetting and washed with 0.9% wt/vol NaCl solution by centrifugation at 700 × g for 15 min. Non-viable cells were identified by staining with trypan blue and cell viability was calculated using the total cell count and the count of non-viable cells. Approximately 1 million PBMCs were transferred in cryovials in 1 ml 90% heat-inactivated fetal calf serum (FCS) and 10% dimethyl sulfoxide (DMSO; Sigma) and immediately placed into a freezing box, placed overnight into an −80 °C freezer. On the following day, the vials were placed at −150 °C for storage.

### Cell-Based Assay for Detection of Antibodies to Cell Surface MOG in Plasma

HEK293A cells transfected with full-length human MOG were used as antigenic substrate in combination with control cells as previously described (29). Briefly, these stable MOG cells were used to detect patient plasma Ig by flow cytometry. As a control, non-transfected HEK293 cells were used for each sample. Cells were harvested using PBS containing 0.2 mM EDTA. Cells were rinsed in 2% FCS/PBS, blocked with 10% FCS/PBS. About 150,000 cells were incubated with patient plasma at a 1:2 dilution for 1 h at 4°C. Cells were then washed three times with 2% FCS/PBS and incubated with fluorescein isothiocyanate (FITC) conjugated anti-human immunoglobulin anti-IgG H + L Fab’2 secondary antibody (Kallestad FITC conjugate, Bio-Rad, Marnes-la-Coquettes, France) for 15 min at 4°C. Cells were washed with 2% FCS/PBS, fixed in 2% formaldehyde-PBS and re-suspended in 300 μl PBS before analysis. A total of 50,000 events per sample were recorded on a FACS Canto II instrument. Data analysis was performed using Flow Jo software (Ashland, OR, USA) and Excel. Binding was expressed as mean fluorescence intensity (MFI). Levels of specific antibody binding in transfected cells were expressed as ΔMFI. ΔMFI was determined by the subtraction of MFI obtained with HEK293 control cells from the MFI obtained with HEK293MOG^+^ cells. A ΔMFI greater than mean + 6 standard deviations (SDs) of values of the control patients’ samples was considered positive. Each experiment was performed at least twice. Positive plasmas were further tested at dilutions of 1:10 to 1:640 by serial dilution with a threshold of 1:160 to define MOG-Ab positivity.

### T Lymphocytes Activation and Flow Cytometry

PBMCs were thawed by warming cryovials rapidly in a 37 °C water bath for approximately 1 min until the ice disappeared. The cell suspension was transferred to a 15 ml centrifuge tube and slowly mixed with 7 ml of warmed culture medium. After centrifugation (400 × g for 10 min), the supernatant solution was removed, and cell pellets suspended in 1 ml fresh culture medium. PBMCs (2 × 10^5^ cells/well) were cultured for 44 h (37°C, 5% CO2) in 200 μl DMEM supplemented with 10% FCS and 1% penicillin/streptomycin, with or without 10 μg/ml rh-MOG, or with 5 μg/ml phytohemagglutinin (PHA, Sigma-Aldrich) as a positive stimulation control as described (28, 30). After 44 h of incubation time, Golgi plug (1 μl/ml, BD Biosciences) and Golgi stop (0.67 μl/ml, BD Biosciences) were added to the media in each well and the cultures incubated for another 4 h. Cells were washed and stained to detect antigen-specific CD4^+^ T cell subsets, as previously described (28), using commercial mAbs according to the manufacturer’s guidelines: anti-CD3-BV768 (SP34–2, BD), anti-CD4-BV605 (L200, BD), anti-CD45RA (HI100 PE Cy7, BD)), and anti-FOXP3-APC (236A/E7, BD). Intracellular staining for Foxp3 required permeabilization buffer and the Foxp3 buffer kit (BD) was used following the manufacturer’s instructions. Intracellular staining also included anti-IFN-γ (clone B27, V450, BD), anti-IL-4 (clone 8D4-8, AF488, BD) and anti-IL-17 (clone N49-653, AF700, BD). Cells were analyzed by flow cytometry with BD-LSR Fortessa (BD Biosciences) using BD FACSDiva software, with at least 100,000 events collected. FlowJo software was used for analysis.

### Statistical Analysis

We performed statistical analyses of cellular immunophenotyping using GraphPad Prism version 8.0.1 (GraphPad Software, San Diego, CA, USA). Data are presented as mean ± standard error of the mean (SEM). For all data sets which could be accurately modeled by a Gaussian normal distribution, an unpaired t-test was used for analysis of differences between groups; otherwise, the Mann-Whitney U-test was used. Within each group, paired comparison of non-stimulated *versus* rh-MOG stimulated was performed for normal distribution using the paired t-test; otherwise, the non-parametric Wilcoxon signed rank test was used. Statistical significance was assigned to values of *p* < 0.05, and the symbols used were *p <*0.05 (*), *p <*0.01 (**) and *p <*0.001 (***). For demographic data, one-way ANOVA test, Chi-square test and Fisher’s exact test were used.

## Results

### Demographic Characteristics of Patients

Demographic data are summarized in **Table 1**. Twenty-two children were included in this study. The mean onset age of each group were similar as analyzed by one-way ANOVA, but that of MS was significantly higher than that of MOGR (unpaired t-test *p* = 0.009). The gender proportion of each group was similar as analyzed by Chi-square test, but MOGR occurred only in female in our cohort (5/5, 100%), a proportion significantly higher than that in MOGNR (Fisher’s exact test *p* = 0.027) and in MS (Fisher’s exact test *p* = 0.044).

### Rh-MOG Induced Th2 and Th17 Lymphocytes in MOGNR

**Figure 1:**
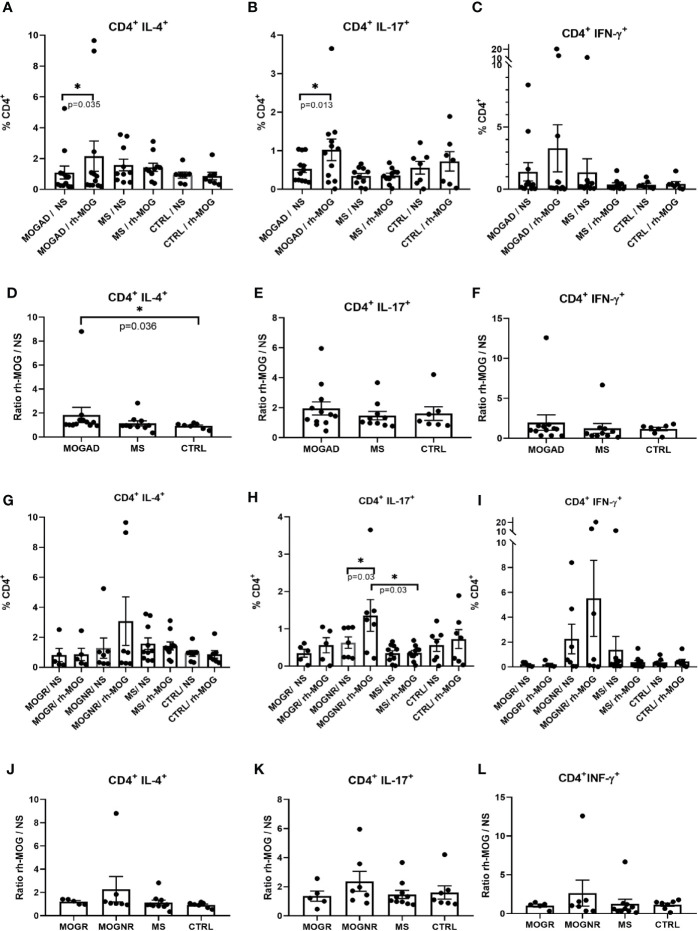
Th2 and Th17 is increased upon rh-MOG stimulation in MOGAD. The percentages of Th2 **(A, G)**, Th17 **(B, H)** and Th1 **(C, I)** cells in the CD4^+^ gate is shown in MOGAD **(A–C)**, or in relapsing ones (MOGR) and non-relapsing ones (MOGNR) **(G–I)**, multiple sclerosis (MS), and controls patients (CTRL), either not stimulated (NS) or stimulated by rh-MOG. Ratio of Th2 **(D, J)**, Th17 **(E, K)** and Th1 **(F, L)** stimulated by rh-MOG *versus* NS are given. Mean and SEM values are indicated as well as *p* values when significant (Wilcoxon for NS *vs.* MOG and Mann-Whitney for groups comparison). *p < 0.05.

The percentage of CD4^+^ Th2 cells (CD3^+^CD4^+^IL-4^+^) was significantly increased after rh-MOG stimulation in the MOGAD group (means: 1.09 ± 0.42 *vs.* 2.15 ± 0.98; Wilcoxon-test *p* = 0.035) while no change was detected in the MS group (means: 1.58 ± 0.38 *vs.* 1.43 ± 0.26; Wilcoxon-test *p* = 0.677) and in the control group (means: 0.92 ± 0.19 *vs.* 0.86 ± 0.25; Wilcoxon-test *p* = 0.687) (**Figure 1A**). The ratio of rh-MOG-stimulated-Th2 cells to unstimulated-ones was significantly higher in the MOGAD group than in the control group (means: 1.83 ± 0.64 *vs.* 0.91 ± 0.8; Mann-Whitney U-test *p* = 0.036) (**Figure 1D**). When comparing MOGNR and MOGR, the percentage of Th2 cell was increased upon rh-MOG stimulation in MOGNR (means: 1.28 ± 0.67 *vs.* 3.07 ± 1.62; Wilcoxon-test *p* = 0.078) but not in MOGR (means: 0.82 ± 0.44 *vs.* 0.86 ± 0.41; Wilcoxon-test *p* = 0.437) (**Figure 1G**). The ratio of rh-MOG-stimulated-Th2 cells to unstimulated-ones was higher in MOGNR than in MOGR without reaching significance (**Figure 1J**).

The percentage of CD4^+^ Th17 lymphocytes (CD3^+^CD4^+^IL-17^+^), was also significantly increased in the MOGAD group when comparing unstimulated to rh-MOG stimulated PBMC (means: 0.53 ± 0.1 *vs.* 1.02 ± 0.28; Wilcoxon-test *p* = 0.013), but not in MS (means: 0.34 ± 0.07 *vs.* 0.35 ± 0.06; Wilcoxon-test *p* = 0.537) nor in the control groups (means: 0.55 ± 0.16 *vs.* 0.72 ± 0.25; Wilcoxon-test *p* = 0.437) (**Figure 1B**). No significant differences were observed when considering the ratio of rh-MOG-stimulated-Th17 cells to unstimulated-ones among the three groups (means: 1.95 ± 0.44; 1.47 ± 0.28 and 1.6 ± 0.45 in MOGAD, MS and control, respectively) (**Figure 1E**). When separating MOGAD into MOGNR and MOGR, it appeared that the Th17 percentages increased significantly upon rh-MOG stimulation in MOGNR (means: 0.63 ± 0.15 *vs.* 1.36 ± 0.43; Wilcoxon-test *p* = 0.03) as well as in MOGR but without reaching significance (means: 0.34 ± 0.1 *vs.* 0.56 ± 0.2; Wilcoxon-test *p* = 0.25) (**Figure 1H**). The ratio of rh-MOG-stimulated-Th17 cells to unstimulated-ones was higher in MOGNR than in MOGR without reaching significance (**Figure 1K**).

Th1 cells (CD3^+^CD4^+^IFN-γ^+^) increased upon rh-MOG stimulation in PBMC from MOGAD patients (means: 1.4 ± 0.74 *vs.* 3.3 ± 1.89; Wilcoxon-test *p* = 0.747) while it decreased in MS patients without reaching significance (means: 1.37± 1.08 *vs.* 0.37 ± 0.14; Wilcoxon-test *p* = 0.232) (**Figure 1C**). The ratio of rh-MOG-stimulated-Th1 cells to unstimulated-ones was higher in MOGAD than in MS (means: 1.97 ± 0.97 *vs.* 1.23 ± 0.61; Mann-Whitney U-test *p* = 0.159) (**Figure 1F**). When MOGAD patients were subdivided in MOGNR and MOGR, we observed that, upon rh-MOG stimulation, the proportion of Th1 cells in MOGNR was increased by 2.4-fold, although not reaching a statistical significance (means: 2.25 ± 1.19 *vs.* 5.51 ± 3.06; Wilcoxon-test *p* = 0.812), whereas it was unchanged in MOGR (means: 0.2 ± 0.07 *vs.* 0.19 ± 0.09; Wilcoxon-test *p* = 0.875) (**Figure 1I**). Accordingly, the ratio of rh-MOG-stimulated-Th1 cells to unstimulated-ones tended to be higher in MOGNR than in MOGR (means: 1.03 ± 0.19 *vs.* 2.64 ± 1.67; Mann-Whitney U-test *p* = 0.755) without reaching significance (**Figure 1L**).

### CD4^+^Foxp3^+^ T_regs_ Increased Upon rh-MOG Stimulation in MOGNR

**Figure 2:**
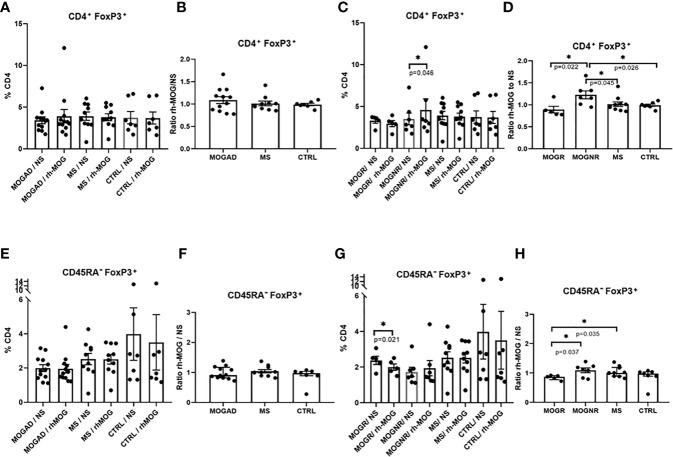
T_regs_ are increased in MOGNR unlike in MOGR. The percentage of T_regs_ (Foxp3^+^) in CD4^+^ gate **(A, C)** and in CD45RA^-^ **(E, G)** are shown in each group of patients, either not stimulated (NS) or stimulated by rh-MOG. Ratio of CD4^+^Foxp3^+^ **(B, D)** and CD45RA^-^Foxp3^+^ **(F, H)** stimulated by rh-MOG *versus* NS are given. Mean and SEM values are indicated as well as *p* values when significant [Wilcoxon for NS *vs.* MOG, Mann-Whitney for groups comparison for all figures except paired t-test for **(F)** and unpaired t-test for **(B, D–F, H)**]. *p < 0.05.

The percentage of regulatory T lymphocytes T_regs_ (CD3^+^CD4^+^Foxp3^+^), was not different when comparing MOGAD, MS and control groups. No significant change in the T_reg_ percentages was observed following the rh-MOG stimulation in MOGAD (means: 3.42 ± 0.41 *vs.* 3.91 ± 0.8; Wilcoxon-test *p* = 0.505), in MS (means: 3.92 ± 0.49 *vs.* 3.78 ± 0.41; Wilcoxon-test *p* = 0.492) and in control patients (means: 3.73 ± 0.74 *vs.* 3.69 ± 0.72; Wilcoxon-test *p* = 0.687) (**Figure 2A**). Accordingly, the ratio of rh-MOG-stimulated-T_reg_ cells to unstimulated-ones was not significantly different among MOGAD, MS, and control groups (**Figure 2B**). Interestingly, when MOGAD patients were separated in two subgroups, there was a significant increase in T_regs_ after rh-MOG stimulation in MOGNR (means: 3.51 ± 0.7 *vs.* 4.59 ± 1.33; Wilcoxon-test *p* = 0.046), but not in MOGR (means: 3.3 ± 0.29 *vs.* 2.95 ± 0.36; Wilcoxon-test *p* = 0.375) (**Figure 2C**). When considering the ratio of rh-MOG-stimulated-T_reg_ cells to unstimulated-ones, it was significantly higher in MOGNR than that in MOGR patients (means: 1.23 ± 0.09 *vs.* 0.89 ± 0.07; unpaired t-test *p* = 0.022) and that in MS (mean: 1 ± 0.05; unpaired t-test *p* = 0.045) and in control patients (mean: 0.95 ± 0.03; unpaired t-test *p* = 0.026) (**Figure 2D**).

### CD45RA^-^Foxp3^+^ T_regs_ Decreased Upon rh-MOG Stimulation in MOGR

We next evaluated the percentage of Foxp3^+^ T_regs_ among CD45RA^-^ cells to evaluate the balance between CD4^+^Foxp3^+^ T_regs_ and effector/memory CD45RA^-^Foxp3^+^ T_reg_ cells. This latter cell percentage did not differ after rh-MOG stimulation in MOGAD (means: 1.91 ± 0.2 *vs.* 1.83 ± 0.26; Wilcoxon-test *p* = 0.294), MS (means: 2.52 ± 0.33 *vs.* 2.51 ± 0.28; Wilcoxon-test *p* = 0.865) and control groups (**Figure 2E**). The same result was observed when expressed as a ratio (ratio of Foxp3^+^ T_reg_ cells among CD45RA^-^ cells in rh-MOG-stimulated *versus* unstimulated conditions) (**Figure 2F**). When subdividing MOGAD into MOGNR and MOGR, it appeared that the percentage of Foxp3^+^ cells in CD45RA^-^ fraction was significantly decreased in the MOGR group after rh-MOG stimulation (means: 2.37 ± 0.23 *vs.* 1.99 ± 0.17; paired t-test *p* = 0.021) but not in the MOGNR group (**Figure 2G**). Interestingly, the ratio of rh-MOG-stimulated-CD45RA^-^Foxp3^+^ T_reg_ cells to unstimulated-ones was significantly lower in MOGR as compared to MS (means: 0.84 ± 0.03 *vs.* 1.04 ± 0.05; unpaired t-test *p* = 0.035), and as compared to MOGNR (mean: 1.1 ± 0.08; unpaired t-test *p* = 0.037). These results indicate a decrease in Foxp3^+^ cells among CD45RA^-^ cells in MOGR after stimulation by rh-MOG (**Figure 2H**).

### T_regs_ Subpopulation Differences Between MOGR and MOGNR

**Figure 3:**
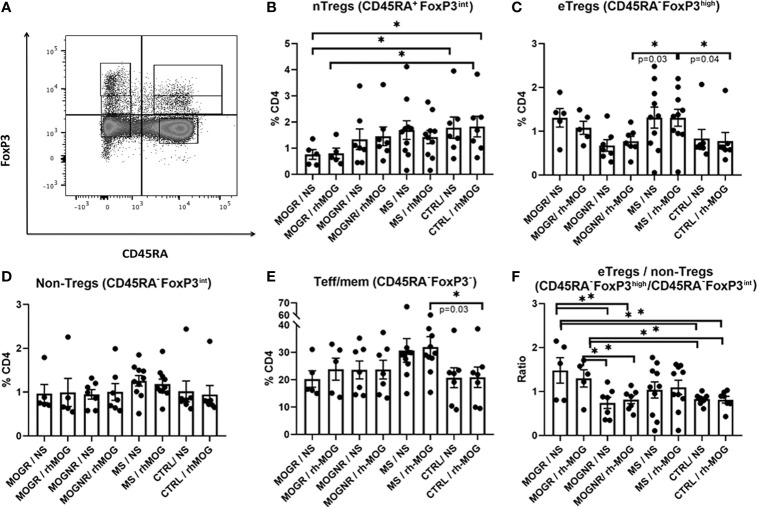
Ratio eT_regs_ to non-T_regs_ is higher in MOGR than in MOGNR. **(A)** Representative dot plot showing gating strategy for Foxp3-expressing subsets. The different Foxp3^+^ subsets were analyzed following the differential expression of CD45RA and Foxp3 staining, gated on CD3^+^CD4^+^. Naive T_regs_ (nT_regs_: CD4^+^CD45RA^+^Foxp3^int^) **(B)** and effector T_regs_ (eT_regs_: CD4^+^CD45RA^-^Foxp3^high^) are both known to be suppressive *in vitro* whereas non-suppressive T_regs_ (non-T_regs_ CD4^+^CD45RA^-^Foxp3^int^) lack suppressive activity and are pro-inflammatory (31). The percentage of eTregs CD45RA^-^Foxp3^high^ **(C)**, of non-T_regs_ CD45RA^-^Foxp3^int^ **(D)** and of T_eff/mem_ CD45RA^-^Foxp3^-^ **(E)** in CD4 gate are shown. In **(F)** ratio of CD45RA^-^Foxp3^high^ to CD45RA^-^Foxp3^-^ (eT_regs_/non-T_regs_) are given. Mean and SEM values are indicated as well as *p* values when significant [Mann-Whitney for groups comparison for all figures except unpaired t-test for **(F)**]. *p < 0.05.

Distinct T_reg_ subsets have been identified based on their phenotypic and functional properties. On the basis of the expression of CD45RA and Foxp3, one can distinguish 3 important Foxp3-expressing subsets: naive T_reg_ cells (nT_regs_: CD4^+^CD45RA^+^FoxP3^int^), effector T_reg_ cells (eT_regs_: CD4^+^CD45RA^-^Foxp3^high^) which are both suppressive, and non-suppressive Foxp3+ cells (non-T_regs_: CD4^+^CD45RA^-^Foxp3^int^). The majority of nT_regs_ are thought to recently originate from the thymus, that may subsequently convert into eT_regs_ (31). This strategy of analysis allows better identification of the Foxp3 expressing cells exhibiting suppressive properties (31). To investigate whether differences in these T_reg_ subsets existed among our groups of patients, we quantified these populations (nT_regs_, eT_regs_ and non-T_regs_ among Foxp3^+^ cells) as well as T effector/memory cells among Foxp3- cells (T_eff/mem_: CD4^+^CD45RA^-^Foxp3^-^). The gating strategy used is presented in **Figure 3A**.

We observed that nT_regs_ tended to be lower in MOGR than in any other group of patients, and significantly lower than in control patients (**Figure 3B**). No change in the percentage of eT_regs_ was observed between MOG-stimulated and unstimulated conditions in MOGNR, MOGR, MS and control groups. However, when comparing the proportions of eT_regs_ upon rh-MOG stimulation, MS exhibited a higher proportion of eT_regs_ than MOGNR and control (**Figure 3C**). This was specific to the eT_regs_ fraction since no difference in the percentages of non-T_regs_ Foxp3^+^ cells among MOGR, MOGNR MS and control, in MOG-stimulated or unstimulated conditions was observed (**Figure 3D**). As a control, we also determined the proportion of T_eff/mem_ Foxp3^-^ cells (CD4^+^CD45RA^-^Foxp3^-^). This fraction was not affected by rh-MOG stimulation as no difference was observed between stimulated and unstimulated conditions. We observed that MS exhibited higher proportion of T_eff/mem_ Foxp3^-^ cells compared to the control group (**Figure 3E**). Lastly, we evaluated the ratio of eT_regs_ Foxp3^+^ cells/non-T_regs_ Foxp3^+^ cells to evaluate the balance between suppressive and effector cells within the activated fraction. The impact of rh-MOG stimulation on this ratio was not significant in all group considered. However, the MOGR showed a significantly higher eT_regs_/non-T_regs_ ratio than MOGNR and control, in both non-stimulated and rh-MOG stimulated conditions (**Figure 3F**).

## Discussion

One main finding of this study is that there are differences in the CD4^+^ T cells immunological phenotypes of ADS clinical subsets, with a significant increase in CD4^+^ Th2 and Th17 cells following stimulation with rh-MOG in MOGAD children at onset of demyelinating events but not in MS and control patients. Within MOGAD patients, a significant increase of Th17 induced by rh-MOG stimulation was observed in patients without relapse (MOGNR). Importantly, CD4^+^Foxp3^+^ T_regs_ were significantly increased in response to rh-MOG in MOGNR, while CD45RA^-^Foxp3^+^ T_regs_ decreased upon rh-MOG stimulation in MOGR patients.

Our results point at a major role of MOG-specific CD4^+^ T cells in MOGAD pathogenesis. The absence of significant changes in MS cells upon rh-MOG stimulation suggests that CD4^+^ lymphocytes do not respond to MOG antigen in MS, reminiscent of the absence of MOG-Abs in this pediatric ADS (9, 10). Several groups also showed no difference in number of circulating MOG-autoreactive T-cells in MS compared to healthy controls (32). However, in a recent study, a novel and highly sensitive method for detection of antigen-specific T-cells using bead-bound MOG as stimulant allowed to detect circulating autoreactive CD4^+^ T-cells producing IFN-γ, IL-22 or IL-17A in 46–59% of adult MS patients. The patients included in this study were adults with MS under natalizumab, which blocks the very late antigen 4 (VLA-4) dependent cell migration across the blood-brain barrier into CNS, and could result in an accumulation of MOG specific T-cells in the circulation of treated patients, that may have increase their numbers in this assay (33) whereas our patients, at onset of the disease, had not been treated yet.

The rates of MOG-specific Th1 lymphocytes are low in our ADS patients as compared to that found in other studies (34). This may be explained by the fact that in this latter study PMA-ionomycin is used to stimulate the cells, whereas we compared rh-MOG stimulated cells to unstimulated ones.

Involvement of Th2 inflammation in autoimmunity including ADS is increasingly found as a component of these diseases (35). In some EAE models, a harmful Th2 response upon exposure to MOG autoantigen was observed beside the classical Th1/Th17 responses in mice (36) and in marmosets (37). These studies suggest that a Th2-type immune response plays a role in the development of EAE, giving further importance to the increase in Th2 cells observed in our MOGAD patients. Recently, in neuromyelitis optica spectrum disorder (NMOSD), an autoimmune demyelinating disorder characterized by auto-Abs targeting the astrocytic aquaporin-4 (AQP4) water channel in the serum of patients, it was found that a therapeutic strategy promoting a shift from Th1/Th17 to Th2 responses is potentially deleterious in NMOSD (35). In other autoimmune diseases with autoantibodies such as systemic lupus erythematosus (SLE), beside the well-established role of Th1/Th17, a Th2 environment and increased basophils are associated with lupus nephritis in human (38).

In rodent EAE, it has also been reported that IL-17-deficient mice have a less severe disease than wild-type mice (39), and that IL-17 worsened EAE in mice (40). In EAE in marmosets, it was found that treatment with an anti-IL-17A antibody induced a moderate delay of clinical scores, without abrogating EAE development (41). Th17 have often been associated with demyelinating diseases in children and adults, such as MS (42, 43) and MOGAD (44). Our results support this finding and suggest that Th17 may be more particularly involved in MOGNR patients where it is significantly increased as compared to MOGR patients.

In MOGNR patients, beside the increase of MOG-specific Th2 and Th17 responses, we observed a significant increase in CD4^+^Foxp3^+^ T_reg_ cells. On the contrary, in MOGR patients, effector/memory CD45RA^-^Foxp3^+^ T_regs_ significantly decreased upon rh-MOG stimulation. Th17 is known to induce autoimmune tissue injury, whereas T_regs_ inhibit autoimmunity and tissue injury. Disruption of the Th17/T_reg_ balance is thought to be involved in the development of various autoimmune disorders and chronic inflammation (45, 46). When we assessed specifically T_regs_, the ratio of CD4^+^Foxp3^+^ T cell percentage upon stimulation *versus* unstimulated conditions was higher in MOGNR compared to MOGR, MS and control. Focusing on the percentage of Foxp3^+^ cells among CD45RA^-^ cells showed the relative contribution of Foxp3^+^ cells among effector/memory cells. Interestingly, we observed a reduced percentage of Foxp3^+^ cells among CD45RA^-^ cells upon rh-MOG-stimulation in MOGR but not MOGNR. Because Foxp3 expressing cells may include cells with different suppressive activity and notably a non-suppressive fraction, we further studied the Foxp3^+^ fraction. In MOGR, nT_regs_ tended to be lower than in any other group of patients. On the contrary, eT_regs_ were higher in MOGR than in MOGNR and control groups. Although the eT_regs_ fraction decreased upon stimulation by rh-MOG in MOGR, the percentages in stimulated conditions in MOGR remained higher than in MOGNR and control group. Foxp3 is essential for differentiation and suppressive function of T_regs_. In human, conventional non T_reg_ T-cells have been shown to transiently express Foxp3 upon activation (47). Additionally, emerging evidence suggests that Foxp3 expression is not always stable in T_regs_ and can be lost. Several studies on T_reg_ in other autoimmune diseases, such as type 1 diabetes and rheumatoid arthritis, suggest a loss of Foxp3 expression and the generation of pathogenic Th17 cells (48), in association with Foxp3 instability in T_regs_ in these diseases. Different mechanisms would be involved in controlling the stability and expression of Foxp3. Demethylation of an evolutionarily conserved element within the Foxp3 genomic locus, conserved noncoding sequences 2 (CNS2), was described as an important feature regarding its expression stability (49, 50). Recently, downregulation of Foxp3 mRNA expression was described in PBMC of NMOSD patients (51). The decrease, significant for CD45RA^-^Foxp3^+^ T_regs_ and trend for eT_regs_, we found in MOGR upon rh-MOG stimulation could also be explained by apoptosis of T_regs_. In healthy condition, T_regs_ are resistant to apoptosis induced by T-cell receptor (TCR) (52), including T_regs_ expressing self-reactive TCR (53). However, in autoimmune thyroiditis, it was recently suggested that apoptosis of T_regs_ through self-reactive TCR activation can drive autoimmunity (54). In a study based on PBMC stimulation *in vitro*, examination of AQP4-specific T-cells revealed a significantly reduced frequency of T_regs_ in NMOSD patients in response to rhAQP4, in comparison to healthy controls (55). Decrease of Foxp3^+^ T_reg_ cells could influence the multiphasic evolution of MOGAD and be linked to a poor control of inflammation. In our study, it is tempting to speculate that MOGNR patients, which have higher Th17 in response to rh-MOG than MOGR at onset, can control autoimmunity since their T_regs_ are also increased upon rh-MOG stimulation. On the contrary, MOGR patients, in which T_regs_ and particularly CD45RA^-^Foxp3^+^ T_regs_ are decreased in response to rh-MOG, may have intermittent loss of tolerance toward this autoantigen and therefore enter into a relapsing form of MOGAD.

In primates treated with rhMOG/IFA, an EAE is induced having immune-inflammatory characteristics closer to MOGAD than to MS (27). Subsequent treatment of these primates with anti-DC-ASGPR-MOG increased their number of MOG-specific CD4^+^CD25^+^FOXP3^+^CD39^+^ T_regs_ as compared to controls. This increase likely precluded EAE seen in the controls (28) and reminded the increase in T_regs_ upon rh-MOG stimulation found in MOGNR patients.

One major limitation of our study is the small sample size of patients including MOGAD, but this is inherent to the fact that ADS are rare diseases. The timing of the samples was not always similar in all patients and there might be temporal modifications of immune cells, which could also have biased our work. The absence of healthy control is also a limitation.

## Conclusions

In conclusion, an increase of Th2 and Th17 after stimulation by rh-MOG was observed in ADS children with MOGAD, particularly in MOGNR. T_regs_ have differences in their subset pattern and behave differentially in MOGR and MOGNR. CD4^+^Foxp3^+^ T cell are increased upon rh-MOG stimulation specifically in MOGNR while in MOGR CD45RA^-^FoxP3^+^ T_regs_ decreased upon rh-MOG stimulation. This may reflect instability or apoptosis of T_regs_ induced by rh-MOG that may subsequently contribute to a probable loss of tolerance toward MOG autoantigen in MOGR which may explain relapses in this recurrent pediatric autoimmune disease. These results suggest that T_regs_ are targets to develop new therapeutic strategies of MOGAD.

## Data Availability Statement

The raw data supporting the conclusions of this article will be made available by the authors, without undue reservation.

## Ethics Statement

The studies involving human participants were reviewed and approved by Commission Nationale de l’Informatique et des Libertés. Written informed consent to participate in this study was provided by the participants’ legal guardian/next of kin.

## Author Contributions

PH, CS, RL and KD conceived and designed the experiments. PH, AC, LG, CL and PC performed the experiments. PH, AC, LG, CL, PC and SH-B-A analyzed and interpreted the data. PH, CB, XM and KD wrote the manuscript. All authors contributed to the article and approved the submitted version.

## Funding

This work was supported by the Institut National de la Santé et de la Recherche Médicale (INSERM) and the Assistance Publique-Hôpitaux de Paris.

## Conflict of Interest

The authors declare that the research was conducted in the absence of any commercial or financial relationships that could be construed as a potential conflict of interest.

## Abbreviations

ADS, acquired demyelinating syndromes; ADEM, acute demyelinating encephalomyelitis; AQP, aquaporin; IL-6, interleukin-6; IL-4, interleukin-4; IL-17, interleukin-17; IFN-γ, interferon-γ; NMOSD, neuromyelitis optica spectrum disorder; MOG, myelin oligodendrocyte glycoprotein; MOGAD, MOG antibody-associated disease; MOGNR, non-relapsing MOGAD; MOGR, relapsing MOGAD; MRI, magnetic resonance imaging; MS, multiple sclerosis; TCR, T-cell receptor.

## References

[1] Absoud M Lim MJ Chong WK De Goede CG Foster K Gunny R. Paediatric Acquired Demyelinating Syndromes: Incidence, Clinical and Magnetic Resonance Imaging Features. Mult Scler (2013) 19(1):76–86. 10.1177/1352458512445944 22516794 PMC3409874

[2] de Mol CL Wong YYM van Pelt ED Ketelslegers IA Bakker DP Boon M. Incidence and Outcome of Acquired Demyelinating Syndromes in Dutch Children: Update of a Nationwide and Prospective Study. J Neurol (2018) 265(6):1310–9. 10.1007/s00415-018-8835-6 PMC5990581 29569176

[3] Langer-Gould A Zhang JL Chung J Yeung Y Waubant E Yao J. Incidence of Acquired CNS Demyelinating Syndromes in a Multiethnic Cohort of Children. Neurology (2011) 77(12):1143–8. 10.1212/WNL.0b013e31822facdd PMC3265045 21865580

[4] Probstel AK Dornmair K Bittner R Sperl P Jenne D Magalhaes S. Antibodies to MOG are Transient in Childhood Acute Disseminated Encephalomyelitis. Neurology (2011) 77(6):580–8. 10.1212/WNL.0b013e318228c0b1 21795651

[5] Gardinier MV Amiguet P Linington C Matthieu JM. Myelin/Oligodendrocyte Glycoprotein is a Unique Member of the Immunoglobulin Superfamily. J Neurosci Res (1992) 33(1):177–87. 10.1002/jnr.490330123 1453482

[6] Brilot F Dale RC Selter RC Grummel V Kalluri SR Aslam M. Antibodies to Native Myelin Oligodendrocyte Glycoprotein in Children With Inflammatory Demyelinating Central Nervous System Disease. Ann Neurol (2009) 66(6):833–42. 10.1002/ana.21916 20033986

[7] Kroepfl JF Viise LR Charron AJ Linington C Gardinier MV. Investigation of Myelin/Oligodendrocyte Glycoprotein Membrane Topology. J Neurochem (1996) 67(5):2219–22. 10.1046/j.1471-4159.1996.67052219.x 8863536

[8] Waters P Woodhall M O’Connor KC Reindl M Lang B Sato DK. MOG Cell-Based Assay Detects non-MS Patients With Inflammatory Neurologic Disease. Neurol Neuroimmunol Neuroinflamm (2015) 2(3):e89. 10.1212/NXI.0000000000000089 25821844 PMC4370386

[9] Ketelslegers IA Van Pelt DE Bryde S Neuteboom RF Catsman-Berrevoets CE Hamann D. Anti-MOG Antibodies Plead Against MS Diagnosis in an Acquired Demyelinating Syndromes Cohort. Mult Scler (2015) 21(12):1513–20. 10.1177/1352458514566666 25662345

[10] Hacohen Y Absoud M Deiva K Hemingway C Nytrova P Woodhall M. Myelin Oligodendrocyte Glycoprotein Antibodies are Associated With a non-MS Course in Children. Neurol Neuroimmunol Neuroinflamm (2015) 2(2):e81. 10.1212/NXI.0000000000000081 25798445 PMC4360800

[11] Machado-Santos J Saji E Troscher AR Paunovic M Liblau R Gabriely G. The Compartmentalized Inflammatory Response in the Multiple Sclerosis Brain is Composed of Tissue-Resident CD8+ T Lymphocytes and B Cells. Brain (2018) 141(7):2066–82. 10.1093/brain/awy151 PMC6022681 29873694

[12] Hochmeister S Gattringer T Asslaber M Stangl V Haindl MT Enzinger C. A Fulminant Case of Demyelinating Encephalitis With Extensive Cortical Involvement Associated With Anti-MOG Antibodies. Front Neurol (2020) 11:31. 10.3389/fneur.2020.00031 32117004 PMC7034704

[13] Pilli D Zou A Tea F Dale RC Brilot F. Expanding Role of T Cells in Human Autoimmune Diseases of the Central Nervous System. Front Immunol (2017) 8:652. 10.3389/fimmu.2017.00652 28638382 PMC5461350

[14] Hohlfeld R Dornmair K Meinl E Wekerle H. The Search for the Target Antigens of Multiple Sclerosis, Part 1: Autoreactive CD4+ T Lymphocytes as Pathogenic Effectors and Therapeutic Targets. Lancet Neurol (2016) 15(2):198–209. 10.1016/S1474-4422(15)00334-8 26724103

[15] Iglesias A Bauer J Litzenburger T Schubart A Linington C--. And B-cell Responses to Myelin Oligodendrocyte Glycoprotein in Experimental Autoimmune Encephalomyelitis and Multiple Sclerosis. Glia (2001) 36(2):220–34. 10.1002/glia.1111 11596130

[16] Lebar R Lubetzki C Vincent C Lombrail P Boutry JM. The M2 Autoantigen of Central Nervous System Myelin, a Glycoprotein Present in Oligodendrocyte Membrane. Clin Exp Immunol (1986) 66(2):423–34. PMC1542532 2434274

[17] Berer K Mues M Koutrolos M Rasbi ZA Boziki M Johner C. Commensal Microbiota and Myelin Autoantigen Cooperate to Trigger Autoimmune Demyelination. Nature (2011) 479(7374):538–41. 10.1038/nature10554 22031325

[18] Bettelli E Baeten D Jager A Sobel RA Kuchroo VK. Myelin Oligodendrocyte Glycoprotein-Specific T and B Cells Cooperate to Induce a Devic-like Disease in Mice. J Clin Invest (2006) 116(9):2393–402. 10.1172/JCI28334 PMC1555670 16955141

[19] Bettelli E Pagany M Weiner HL Linington C Sobel RA Kuchroo VK. Myelin Oligodendrocyte Glycoprotein-Specific T Cell Receptor Transgenic Mice Develop Spontaneous Autoimmune Optic Neuritis. J Exp Med (2003) 197(9):1073–81. 10.1084/jem.20021603 PMC2193967 12732654

[20] Krishnamoorthy G Lassmann H Wekerle H Holz A. Spontaneous Opticospinal Encephalomyelitis in a Double-Transgenic Mouse Model of Autoimmune T Cell/B Cell Cooperation. J Clin Invest (2006) 116(9):2385–92. 10.1172/JCI28330 PMC1555668 16955140

[21] Mayer MC Meinl E. Glycoproteins as Targets of Autoantibodies in CNS Inflammation: MOG and More. Ther Adv Neurol Disord (2012) 5(3):147–59. 10.1177/1756285611433772 PMC3349079 22590479

[22] Mader S Gredler V Schanda K Rostasy K Dujmovic I Pfaller K. Complement Activating Antibodies to Myelin Oligodendrocyte Glycoprotein in Neuromyelitis Optica and Related Disorders. J Neuroinflamm (2011) 8:184. 10.1186/1742-2094-8-184 PMC3278385 22204662

[23] Peschl P Schanda K Zeka B Given K Bohm D Ruprecht K. Human Antibodies Against the Myelin Oligodendrocyte Glycoprotein can Cause Complement-Dependent Demyelination. J Neuroinflamm (2017) 14(1):208. 10.1186/s12974-017-0984-5 PMC5657084 29070051

[24] Piddlesden SJ Lassmann H Zimprich F Morgan BP Linington C. The Demyelinating Potential of Antibodies to Myelin Oligodendrocyte Glycoprotein is Related to Their Ability to Fix Complement. Am J Pathol (1993) 143(2):555–64. PMC1887024 7688186

[25] Dale RC Tantsis EM Merheb V Kumaran RY Sinmaz N Pathmanandavel K. Antibodies to MOG Have a Demyelination Phenotype and Affect Oligodendrocyte Cytoskeleton. Neurol Neuroimmunol Neuroinflamm (2014) 1(1):e12. 10.1212/NXI.0000000000000012 25340056 PMC4202678

[26] Marta CB Taylor CM Coetzee T Kim T Winkler S Bansal R. Antibody Cross-Linking of Myelin Oligodendrocyte Glycoprotein Leads to its Rapid Repartitioning Into Detergent-Insoluble Fractions, and Altered Protein Phosphorylation and Cell Morphology. J Neurosci (2003) 23(13):5461–71. 10.1523/JNEUROSCI.23-13-05461.2003 PMC6741276 12843245

[27] Serguera C Stimmer L Fovet CM Horellou P Contreras V Tchitchek N. Anti-MOG Autoantibodies Pathogenicity in Children and Macaques Demyelinating Diseases. J Neuroinflamm (2019) 16(1):244. 10.1186/s12974-019-1637-7 PMC6884758 31785610

[28] Fovet CM Stimmer L Contreras V Horellou P Hubert A Seddiki N. Intradermal Vaccination Prevents anti-MOG Autoimmune Encephalomyelitis in Macaques. EBioMedicine (2019) 47:492–505. 10.1016/j.ebiom.2019.08.052 31492559 PMC6796575

[29] Horellou P Wang M Keo V Chretien P Serguera C Waters P. Increased Interleukin-6 Correlates With Myelin Oligodendrocyte Glycoprotein Antibodies in Pediatric Monophasic Demyelinating Diseases and Multiple Sclerosis. J Neuroimmunol (2015) 289:1–7. 10.1016/j.jneuroim.2015.10.002 26616865

[30] Zaunders JJ Munier ML Seddiki N Pett S Ip S Bailey M. High Levels of Human Antigen-Specific CD4+ T Cells in Peripheral Blood Revealed by Stimulated Coexpression of CD25 and CD134 (Ox40). J Immunol (2009) 183(4):2827–36. 10.4049/jimmunol.0803548 19635903

[31] Miyara M Yoshioka Y Kitoh A Shima T Wing K Niwa A. Functional Delineation and Differentiation Dynamics of Human CD4+ T Cells Expressing the FoxP3 Transcription Factor. Immunity (2009) 30(6):899–911. 10.1016/j.immuni.2009.03.019 19464196

[32] Elong Ngono A Pettre S Salou M Bahbouhi B Soulillou JP Brouard S. Frequency of Circulating Autoreactive T Cells Committed to Myelin Determinants in Relapsing-Remitting Multiple Sclerosis Patients. Clin Immunol (2012) 144(2):117–26. 10.1016/j.clim.2012.05.009 22717772

[33] Bronge M Ruhrmann S Carvalho-Queiroz C Nilsson OB Kaiser A Holmgren E. Myelin Oligodendrocyte Glycoprotein Revisited-Sensitive Detection of MOG-specific T-Cells in Multiple Sclerosis. J Autoimmun (2019) 102:38–49. 10.1016/j.jaut.2019.04.013 31054941

[34] Brucklacher-Waldert V Stuerner K Kolster M Wolthausen J Tolosa E. Phenotypical and Functional Characterization of T Helper 17 Cells in Multiple Sclerosis. Brain (2009) 132(12):3329–41. 10.1093/brain/awp289 19933767

[35] Costanza M. Type 2 Inflammatory Responses in Autoimmune Demyelination of the Central Nervous System: Recent Advances. J Immunol Res (2019) 2019:4204512. 10.1155/2019/4204512 31205957 PMC6530110

[36] Pedotti R Mitchell D Wedemeyer J Karpuj M Chabas D Hattab EM. An Unexpected Version of Horror Autotoxicus: Anaphylactic Shock to a Self-Peptide. Nat Immunol (2001) 2(3):216–22. 10.1038/85266 11224520

[37] Genain CP Abel K Belmar N Villinger F Rosenberg DP Linington C. Late Complications of Immune Deviation Therapy in a Nonhuman Primate. Science (1996) 274(5295):2054–7. 10.1126/science.274.5295.2054 8953031

[38] Charles N Hardwick D Daugas E Illei GG Rivera J. Basophils and the T Helper 2 Environment can Promote the Development of Lupus Nephritis. Nat Med (2010) 16(6):701–7. 10.1038/nm.2159 PMC2909583 20512127

[39] Komiyama Y Nakae S Matsuki T Nambu A Ishigame H Kakuta S. Il-17 Plays an Important Role in the Development of Experimental Autoimmune Encephalomyelitis. J Immunol (2006) 177(1):566–73. 10.4049/jimmunol.177.1.566 16785554

[40] Haak S Croxford AL Kreymborg K Heppner FL Pouly S Becher B. Il-17A and IL-17F do Not Contribute Vitally to Autoimmune Neuro-Inflammation in Mice. J Clin Invest (2009) 119(1):61–9. 10.1172/JCI35997 PMC2613466 19075395

[41] t Hart BA. Experimental Autoimmune Encephalomyelitis in the Common Marmoset: A Translationally Relevant Model for the Cause and Course of Multiple Sclerosis. Primate Biol (2019) 6(1):17–58. 10.5194/pb-6-17-2019 32110715 PMC7041540

[42] Kebir H Ifergan I Alvarez JI Bernard M Poirier J Arbour N. Preferential Recruitment of Interferon-Gamma-Expressing TH17 Cells in Multiple Sclerosis. Ann Neurol (2009) 66(3):390–402. 10.1002/ana.21748 19810097

[43] Vargas-Lowy D Kivisakk P Gandhi R Raddassi K Soltany P Gorman MP. Increased Th17 Response to Myelin Peptides in Pediatric MS. Clin Immunol (2013) 146(3):176–84. 10.1016/j.clim.2012.12.008 23352968

[44] Kothur K Wienholt L Tantsis EM Earl J Bandodkar S Prelog K. B Cell, Th17, and Neutrophil Related Cerebrospinal Fluid Cytokine/Chemokines are Elevated in MOG Antibody Associated Demyelination. PloS One (2016) 11(2):e0149411. 10.1371/journal.pone.0149411 26919719 PMC4769285

[45] Nie H Zheng Y Li R Guo TB He D Fang L. Phosphorylation of FOXP3 Controls Regulatory T Cell Function and is Inhibited by TNF-alpha in Rheumatoid Arthritis. Nat Med (2013) 19(3):322–8. 10.1038/nm.3085 23396208

[46] Bettelli E Carrier Y Gao W Korn T Strom TB Oukka M. Reciprocal Developmental Pathways for the Generation of Pathogenic Effector TH17 and Regulatory T Cells. Nature (2006) 441(7090):235–8. 10.1038/nature04753 16648838

[47] Pillai V Ortega SB Wang CK Karandikar NJ. Transient Regulatory T-cells: A State Attained by All Activated Human T-Cells. Clin Immunol (2007) 123(1):18–29. 10.1016/j.clim.2006.10.014 17185041 PMC1868523

[48] Komatsu N Okamoto K Sawa S Nakashima T Oh-hora M Kodama T. Pathogenic Conversion of Foxp3+ T Cells Into TH17 Cells in Autoimmune Arthritis. Nat Med (2014) 20(1):62–8. 10.1038/nm.3432 24362934

[49] Floess S Freyer J Siewert C Baron U Olek S Polansky J. Epigenetic Control of the Foxp3 Locus in Regulatory T Cells. PloS Biol (2007) 5(2):e38. 10.1371/journal.pbio.0050038 17298177 PMC1783672

[50] Polansky JK Kretschmer K Freyer J Floess S Garbe A Baron U. DNA Methylation Controls Foxp3 Gene Expression. Eur J Immunol (2008) 38(6):1654–63. 10.1002/eji.200838105 18493985

[51] Brill L Lavon I Vaknin-Dembinsky A. Foxp3+ Regulatory T Cells Expression in Neuromyelitis Optica Spectrum Disorders. Mult Scler Relat Disord (2019) 30:114–8. 10.1016/j.msard.2019.01.047 30771576

[52] Taylor SR Alexander DR Cooper JC Higgins CF Elliott JI. Regulatory T Cells are Resistant to Apoptosis Via TCR But Not P2X7. J Immunol (2007) 178(6):3474–82. 10.4049/jimmunol.178.6.3474 17339442

[53] Fassett MS Jiang W D’Alise AM Mathis D Benoist C. Nuclear Receptor Nr4a1 Modulates Both Regulatory T-cell (Treg) Differentiation and Clonal Deletion. Proc Natl Acad Sci U S A (2012) 109(10):3891–6. 10.1073/pnas.1200090109 PMC3309794 22345564

[54] Badami E Cexus ONF Quaratino S. Activation-Induced Cell Death of Self-Reactive Regulatory T Cells Drives Autoimmunity. Proc Natl Acad Sci U S A (2019) 116(52):26788–97. 10.1073/pnas.1910281116 PMC6936679 31818938

[55] Varrin-Doyer M Spencer CM Schulze-Topphoff U Nelson PA Stroud RM Cree BA. Aquaporin 4-Specific T Cells in Neuromyelitis Optica Exhibit a Th17 Bias and Recognize Clostridium ABC Transporter. Ann Neurol (2012) 72(1):53–64. 10.1002/ana.23651 22807325 PMC3405197

